# Individual and additive effects of vitamin D, omega-3 and exercise on DNA methylation clocks of biological aging in older adults from the DO-HEALTH trial

**DOI:** 10.1038/s43587-024-00793-y

**Published:** 2025-02-03

**Authors:** Heike A. Bischoff-Ferrari, Stephanie Gängler, Maud Wieczorek, Daniel W. Belsky, Joanne Ryan, Reto W. Kressig, Hannes B. Stähelin, Robert Theiler, Bess Dawson-Hughes, René Rizzoli, Bruno Vellas, Laure Rouch, Sophie Guyonnet, Andreas Egli, E. John Orav, Walter Willett, Steve Horvath

**Affiliations:** 1 Department of Geriatrics and Aging Research, University of Zurich, Zurich, Switzerland; 2 Research Centre on Aging and Mobility, University of Zurich, Zurich, Switzerland; 3 Department of Aging Medicine Felix-Platter, University of Basel, Basel, Switzerland; 4 Department of Epidemiology, Butler Columbia Aging Center, Mailman School of Public Health, Columbia University, New York, NY USA; 5 Biological Neuropsychiatry & Dementia Unit, School of Public Health and Preventive Medicine, Monash University, Melbourne, Victoria Australia; 6 University Department of Geriatric Medicine Felix Platter, University of Basel, Basel, Switzerland; 7 Department of Geriatrics, University of Basel, Basel, Switzerland; 8 Bone Metabolism Laboratory, Jean Mayer USDA Human Nutrition Research Center on Aging, Tufts University, Boston, MA USA; 9 Division of Bone Diseases, Geneva University Hospitals and Faculty of Medicine, Geneva, Switzerland; 10 IHU HealthAge, Toulouse, France; 11 Institut du Vieillissement, Centre Hospitalo-Universitaire de Toulouse, Toulouse, France; 12 CERPOP UMR1295, University of Toulouse III, Inserm, UPS, Toulouse, France; 13 University Paul Sabatier Toulouse III, Toulouse, France; 14 Department of Pharmacy, Toulouse University Hospitals, Purpan Hospital, Toulouse, France; 15 Department of Health Policy and Management, Harvard University T.H. Chan School of Public Health, Boston, MA USA; 16 Harvard Medical School, Boston, MA USA; 17 Department of Medicine, Brigham and Women’s Hospital, Boston, MA USA; 18 Departments of Nutrition and Epidemiology, Harvard TH Chan School of Public Health, Harvard University, Boston, MA USA; 19 Altos Labs Limited, Cambridge, UK

**Keywords:** Predictive markers, Methylation analysis, Ageing

## Abstract

While observational studies and small pilot trials suggest that vitamin D, omega-3 and exercise may slow biological aging, larger clinical trials testing these treatments individually or in combination are lacking. Here, we report the results of a post hoc analysis among 777 participants of the DO-HEALTH trial on the effect of vitamin D (2,000 IU per day) and/or omega-3 (1 g per day) and/or a home exercise program on four next-generation DNA methylation (DNAm) measures of biological aging (PhenoAge, GrimAge, GrimAge2 and DunedinPACE) over 3 years. Omega-3 alone slowed the DNAm clocks PhenoAge, GrimAge2 and DunedinPACE, and all three treatments had additive benefits on PhenoAge. Overall, from baseline to year 3, standardized effects ranged from 0.16 to 0.32 units (2.9–3.8 months). In summary, our trial indicates a small protective effect of omega-3 treatment on slowing biological aging over 3 years across several clocks, with an additive protective effect of omega-3, vitamin D and exercise based on PhenoAge.

## Abstract (web summary)

Applying epigenetic clocks to samples from the DO-HEALTH trial, Bischoff-Ferrari et al. report a small protective effect of omega-3 supplementation over 3 years on several clocks and an additive protective effect of omega-3, vitamin D and exercise using PhenoAge.

## Main

Epigenetic clocks are DNA methylation (DNAm) algorithms that combine information from measurements across the genome to quantify variations in biological versus chronological aging^1^. Continuous advancements have led to the development of first-^2,3^, second-^4,5^ and third-generation^6^ clocks. Many DNAm clocks have been associated with age-related morbidity and mortality^7^. However, evidence of an association with morbidity and mortality as well as lifestyle is the strongest for the second-generation clocks^4,5,8^ and the third-generation clock DunedinPACE^9^. Prior observational and small clinical studies have linked each of the treatments tested in the DO-HEALTH trial (vitamin D^10,11^, omega-3 (refs. ^5,12–15^) and exercise^16–21^) to modulation of epigenetic clock measures of aging and omega-3 to DNAm changes. The goal of our analysis was to test the hypothesis that vitamin D supplementation, omega-3 supplementation and a simple home exercise program (SHEP), individually and in combination, would slow biological aging in a larger clinical trial. In the DO-HEALTH trial including all 2,157 participants, we reported that omega-3 alone reduced the rate of infections by 13% (ref. ^22^) and the rate of falls by 10% (ref. ^23^), and all three interventions combined had a significant additive benefit on reducing prefrailty by 39% (ref. ^24^) and incident invasive cancer by 61% (ref. ^25^) over a 3-year follow-up.

The DO-HEALTH Bio-Age trial included 777 of the 2,157 DO-HEALTH participants with DNAm measures at baseline and 3 years. DO-HEALTH was a multicenter randomized controlled trial designed to support healthy longevity. It enrolled 2,157 generally healthy and active adults aged 70 years and older across five countries in Europe^22,26^. DO-HEALTH was designed as a 2 × 2 × 2 factorial study testing the effects of vitamin D (2,000 IU per day), omega-3 (1 g per day) and SHEP (three times 30 min per week) individually and in combination over an intervention period of 3 years. Participants were randomized to one of eight treatment arms, and all were followed up with yearly examinations and in-person telephone calls every 3 months. Blood was collected at baseline and 1, 2 and 3 years of follow-up, and DNA was extracted and biobanked.

**Fig. 1:**
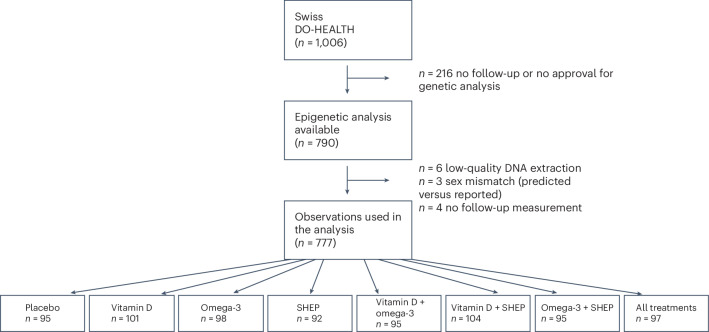
Flowchart of the DO-HEALTH Bio-Age trial in the Swiss subset of DO-HEALTH. The flowchart shows the allocation of participants across the eight treatment arms.

The Swiss National Foundation funded DNAm assays of samples collected at baseline and after 3 years from the Swiss subset of participants. Of the 1,006 Swiss participants in the trial, 777 provided consent for these analyses and had samples available after the application of the exclusion criteria. This group of individuals formed our analysis sample, which had the following characteristics: 59% were women; the mean age at baseline was 75 years; 30% had 25-hydroxyvitamin D (25(OH)D) levels of <20 ng ml^−1^; 53% were healthy agers as defined in the Nurses’ Health Study^27^ (free of major chronic diseases, disabilities, cognitive impairments and mental health limitations); and 88% were physically active (29% were active one to three times per week, and 59% were active more than three times per week). The participant characteristics and allocation across treatment arms in DO-HEALTH are presented in Table 1 and Fig. 1. The Swiss participant subgroup represents a healthier and more active subgroup within the total DO-HEALTH population^28^ (Extended Data Table 1). When Swiss participants with DNAm data were compared to Swiss participants without DNAm data, those without DNAm data were slightly older and slightly less educated but otherwise similar in all covariates assessed (Extended Data Table 1).

**Table 1:** Baseline characteristics of the study population overall and by treatment group

Characteristic	All	Vitamin D	No vitamin D	Omega-3	No omega-3	SHEP	No SHEP
	(*n* = 777)	(*n* = 397)	(*n* = 380)	(*n* = 385)	(*n* = 392)	(*n* = 388)	(*n* = 389)
Chronological age (years), mean (s.d.)	75.47 (4.47)	75.55 (4.54)	75.39 (4.40)	75.14 (4.20)	75.80 (4.70)	75.44 (4.48)	75.50 (4.46)
Female sex, *n* (%)	464 (59.7)	231 (58.2)	233 (61.3)	231 (60.0)	233 (59.4)	233 (60.1)	231 (59.4)
BMI (kg m^−2^), mean (s.d.)	25.72 (4.04)	25.81 (4.13)	25.63 (3.96)	25.74 (3.94)	25.70 (4.15)	25.65 (3.96)	25.80 (4.13)
Years of education, mean (s.d.)	13.48 (3.50)	13.59 (3.64)	13.37 (3.34)	13.50 (3.31)	13.47 (3.67)	13.51 (3.45)	13.46 (3.54)
Healthy ager (NHS criteria), *n* (%)	403 (52.3)	212 (53.9)	191 (50.5)	211 (55.2)	192 (49.4)	200 (51.9)	203 (52.6)
Sangha comorbidity score^60^ (0–30 points), mean (s.d.)	2.66 (2.58)	2.66 (2.54)	2.66 (2.63)	2.57 (2.63)	2.74 (2.53)	2.64 (2.65)	2.68 (2.51)
25(OH)D <20 ng ml^−1^, *n* (%)	263 (33.8)	132 (33.2)	131 (34.5)	123 (31.9)	140 (35.7)	119 (30.7)	144 (37.0)
25(OH)D (ng ml^−1^), mean (s.d.)	23.62 (8.44)	23.76 (8.56)	23.47 (8.31)	23.80 (8.54)	23.44 (8.33)	24.17 (8.53)	23.07 (8.32)
Blood omega-3s (DHA + EPA) <100 ng ml^−1^, *n* (%)	296 (38.1)	152 (38.3)	144 (37.9)	144 (37.4)	152 (38.8)	140 (36.1)	156 (40.1)
Blood omega-3s (DHA + EPA) (ng ml^−1^), mean (s.d.)	94.32 (40.12)	93.40 (40.10)	95.29 (40.17)	94.63 (39.99)	94.02 (40.30)	92.25 (38.23)	96.39 (41.87)
Physical activity, *n* (%)							
Inactive	93 (12.0)	50 (12.6)	43 (11.3)	45 (11.7)	48 (12.2)	38 (9.8)	55 (14.1)
1–3 times per week	227 (29.2)	122 (30.7)	105 (27.6)	99 (25.7)	128 (32.7)	114 (29.4)	113 (29.0)
>3 times per week	457 (58.8)	225 (56.7)	232 (61.1)	241 (62.6)	216 (55.1)	236 (60.8)	221 (56.8)

NHS, Nurses’ Health Study.

**Extended Data Fig. 1:**
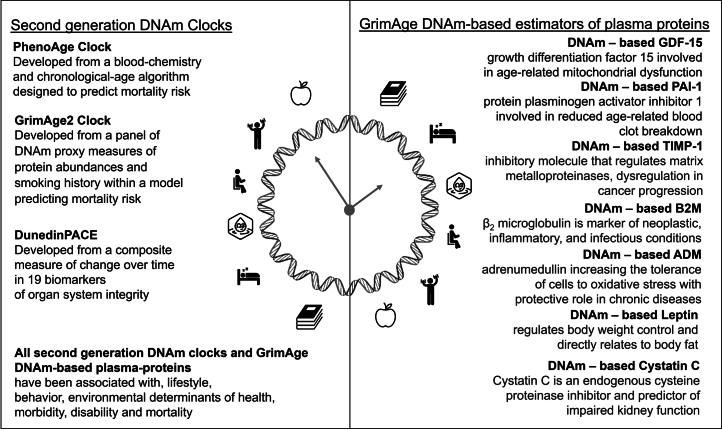
Second generation clocks, Pace of Aging and GrimAge-based plasma proteins explained. Overview of life-style factors associated with second generation DNAm biology of aging measures^5,6,8^ and DNA methylation based surrogate markers of plasma proteins. While DNAm clocks measure biological aging, surrogate proteins are ordinal/quantitative measures, where high values correspond to a higher hazard of mortality^5,8^. For more details and references on the DNAm clocks see Extended Data Table 5. Icons from Noun Project (https://thenounproject.com/).

We focused the primary hypothesis testing on three ‘second-generation’ epigenetic clocks developed from analyses of mortality risk (PhenoAge^4^, GrimAge (ref. ^5^) and GrimAge2 (ref. ^8^)) and a later-generation epigenetic clock, also described as a ‘third-generation’ clock, developed from an analysis of longitudinal change in organ system integrity (DunedinPACE^6^). To enable comparison across studies, we also report results for ‘first-generation’ epigenetic clocks developed from analyses of age differences in DNAm (Horvath^2^, Hannum^3^). As a secondary analysis, we report results from an analysis of the seven DNAm-based surrogate markers of plasma proteins underlying GrimAge, which are quantitative measures in which high values correspond to a higher hazard of mortality^5,8^. We also explore these proteins as they are known to be more responsive to metabolic dysregulation^8^. The epigenetic clocks and DNAm-based protein biomarkers are detailed in Extended Data Fig. 1 (ref. ^5^).

All DNAm-based biomarkers but GrimAge2 and DunedinPACE are based on the principal component (PC) approach. PC versions were computed according to the method described by Higgins-Chen et al.^29^. For the Horvath, Hannum, PhenoAge and GrimAge clocks and the seven DNAm-based surrogate markers of plasma proteins underlying GrimAge, we analyzed versions constructed from DNAm PCs, which can offer superior technical reliability compared to the original versions of these measures^29^. For GrimAge2 and DunedinPACE, we used the original versions as they already demonstrate high technical reliability^5,6,8^.

**Extended Data Fig. 2:**
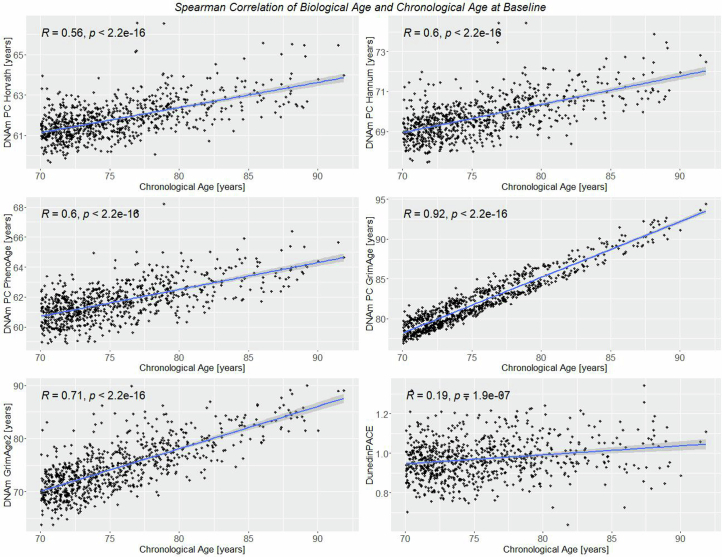
Correlation of chronological age and biological age for DNAm clocks and Pace of Aging. All DNAm measures of age correlated with chronological age at baseline in DO-HEALTH including the pan tissue Horvath clock assessed with Spearman’s rank correlation coefficient. (Spearman’s rank correlation coefficient r = 0.56,), Hannum (r = 0.6), PhenoAge (r = 0.6), GrimAge (r = 0.92), and GrimAge2 (r = 0.71). The correlation between DunedinPACE and chronological age was substantially weaker (r = 0.19).

Mean values of the DNAm measures of biological aging overall and by treatment at baseline and 3 years of follow-up are reported in Extended Data Table 2. Epigenetic clock associations with chronological age at baseline are shown in Extended Data Fig. 2. All clocks correlated with chronological age (PhenoAge *r* = 0.60, GrimAge *r* = 0.92, GrimAge2 *r* = 0.71, DunedinPACE *r* = 0.19). All clocks except for DunedinPACE provide estimates of biological age. For analysis, the biological age values were regressed on chronological age, and the residual values, sometimes referred to as ‘age acceleration’, were standardized and compared between baseline and year 3. DunedinPACE is a measure of the pace of aging; therefore, no residualization is needed as DunedinPACE was developed in a cohort in which all participants were of the same chronological age. The other clocks measure biological age and take on values similar to chronological age. For these clocks, residualization is needed to quantify how much older or younger a person is biologically relative to their chronological age. In contrast, DunedinPACE measures the rate of biological change with aging and takes on values centered around 1, representing 1 year of biological change per calendar year. Because this rate can be compared directly between people of different ages, no residualization is needed.

The analysis compared the change from baseline to the 3-year follow-up in the treatment groups to the change in the control group, using analysis of covariance. The model outcomes were the standardized change scores of age acceleration between the 3-year follow-up values and baseline values. Models included covariates for chronological age (continuous and spline at 85 years), sex, history of falls before study enrollment (a stratifying variable of the trial), body mass index (BMI) and study site.

**Fig. 2:**
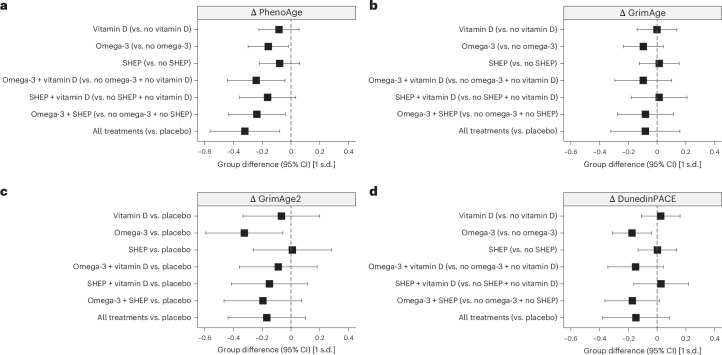
Treatment effects of vitamin D, omega-3 and SHEP individually and in combination on changes in DNAm measures from baseline to year 3. **a**–**d**, Treatment effects are expressed as standardized estimates of the change in DNAm measures from baseline to year 3 at the respective 95% CI. For the PhenoAge (**a**) and GrimAge (**b**) clocks, we analyzed versions constructed from DNAm PCs, which have superior technical reliability compared to the original versions of these measures^29^. For GrimAge2 (**c**) and DunedinPACE (**d**), we used the original versions as they already demonstrate high technical reliability^5,6,8^. All analyses were done in samples of *n* = 777 participants sampled at baseline and the 3-year follow-up, without technical replicates. In **a**–**d**, estimates and 95% CIs from analyses of covariance adjusted for chronological age (continuous + spline at 85 years), sex, falls before the study, BMI, study site and the respective baseline biological age measures are shown. The graphs show the main effects under the assumption of additive effects between treatments in the 2 × 2 × 2 factorial trial design; that is, comparing all individuals treated with vitamin D across the eight treatment arms of the trial to those who did not receive vitamin D (same for omega-3 and SHEP compared to control SHEP). Only for GrimAge2 (**c**) is each treatment arm compared to placebo because of treatment interactions. Consistent effects of omega-3 were observed in decelerating biological aging as measured by PhenoAge (**a**), GrimAge2 (**c**) and DunedinPACE (**d**). PhenoAge additionally showed an additive benefit between treatments, as observed for the clinical outcomes incident prefrailty^24^ and incident invasive cancer^25^ in the same trial. Detailed findings are shown in Extended Data Table 3. For second-generation clocks, standardized units can be converted to months of age retardation using the following equation: (standardized estimate × s.d. + mean) × 12. Overall, the standardized treatment effects from baseline to year 3 were small, ranging from 0.16 to 0.32 units (2.9–3.8 months).

Daily omega-3 supplementation reduced the age-acceleration or pace-of-aging values of three of four clocks in our primary analysis (PhenoAge difference (*d*) = −0.16 (95% confidence interval (CI) −0.02 to −0.30); GrimAge2 *d* = −0.32 (−0.06 to −0.59); DunedinPACE *d* = −0.17 (−0.04 to −0.31)). Vitamin D supplementation and SHEP were not associated with changes in any of the clocks. However, for PhenoAge, there was evidence of additive treatment effects for combinations of omega-3 supplementation with the other two interventions individually and together (range of values of *d*: −0.24 to −0.32). No additive effects across interventions were observed for GrimAge, GrimAge2 or DunedinPACE. No effect of intervention was observed for the first-generation clocks. Treatment effects are shown in Fig. 2 and numerically in Extended Data Table 3.

**Fig. 3:**
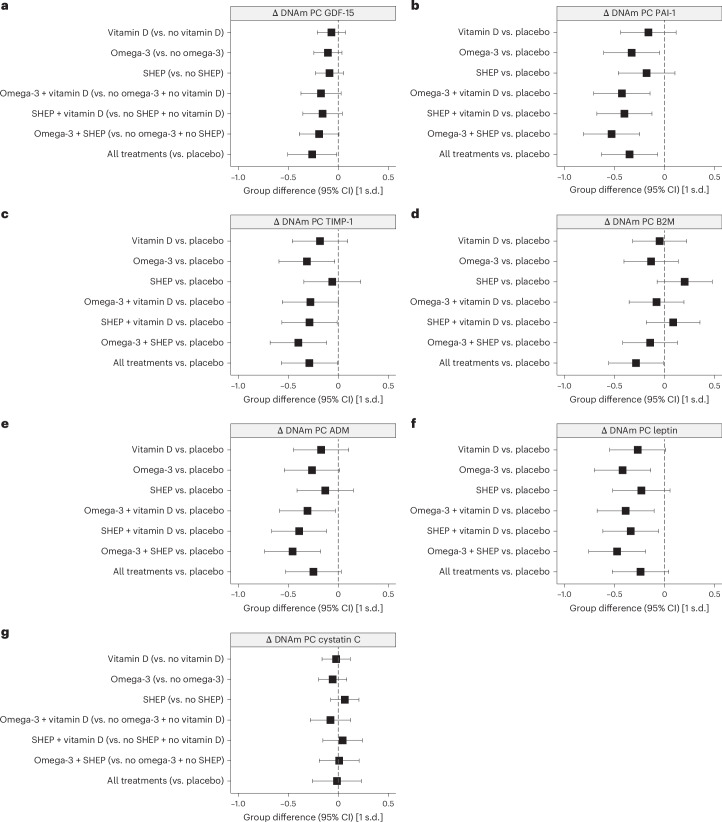
Treatment effects of vitamin D, omega-3 and SHEP individually and in combination on changes in DNAm-based surrogate biomarkers of plasma proteins based on GrimAge. **a**–**g**, For the seven DNAm-based surrogate markers of plasma proteins underlying GrimAge, we analyzed versions constructed from DNAm PCs. The seven DNAm-based biomarkers estimate the abundance of GDF-15 (**a**), PAI-1 (**b**), TIMP-1 (**c**), B2M (**d**), adrenomedullin (ADM; **e**), leptin (**f**) and cystatin C (**g**). All analyses were done in samples of *n* = 777 participants sampled at baseline and the 3-year follow-up, without technical replicates. In **a**–**g**, estimates and 95% CIs from analyses of covariance adjusted for chronological age (continuous + spline at 85 years), sex, falls before the study, BMI, study site and the respective baseline DNAm-based biomarkers are shown. Omega-3 stands out as an individual treatment with a decline in three of the seven DNAm-based surrogate biomarkers of plasma proteins (PAI-1, leptin and TIMP-1). However, the data also show a consistent additive benefit of combining two or all three treatments for several plasma proteins (PAI-1, B2M, TIMP-1 and GDF-15). Detailed findings are shown in Extended Data Table 4.

GrimAge can be interpreted as a linear combination of DNAm-based surrogate markers of plasma proteins. Some of these are known to be more responsive to metabolic dysregulation^8^. DNAm-based plasminogen activation inhibitor 1 (PAI-1), leptin and tissue inhibitor metalloproteinase 1 (TIMP-1) were modified by omega-3 supplementation (range of values of *d* = −0.31 to −0.42), and PAI-1, β2-microglobulin (B2M), TIMP-1 and growth differentiation factor 15 (GDF-15) all showed evidence of modification by combinations of interventions (range of values of *d* = −0.26 to −0.53). Treatment effects are shown in Fig. 3 and numerically in Extended Data Table 4.

**Extended Data Fig. 3:**
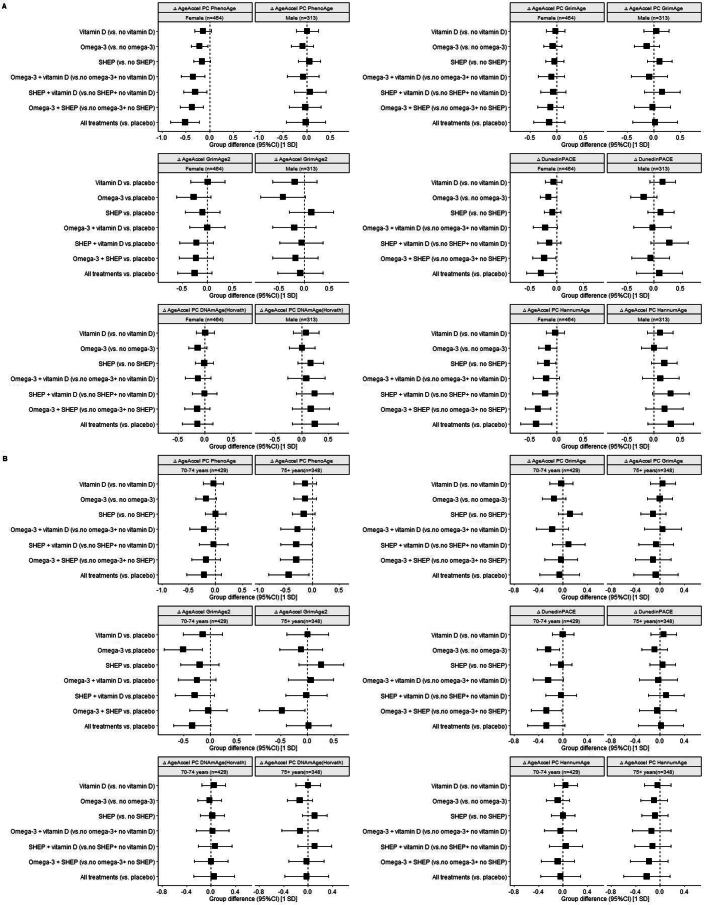
Stratified treatment effects of vitamin D, omega-3 and SHEP (simple home exercise program) individually and in combination on change of DNAm measures from baseline to year 3. Panels **A** and **B** show regression coefficients, and their 95% CI derived from analysis of covariance adjusted for chronological age (continuous + spline at 85 years), sex, falls before study, body mass index, study site, respective baseline biological age measure. All analyses were done in n = 777 participants sampled at baseline and at 3-year follow-up, without technical replicates. The treatment effects in Panel A are stratified by biological sex (male vs. female) and Panel B by chronological age (70-74 vs 75+ years).

**Extended Data Fig. 4:**
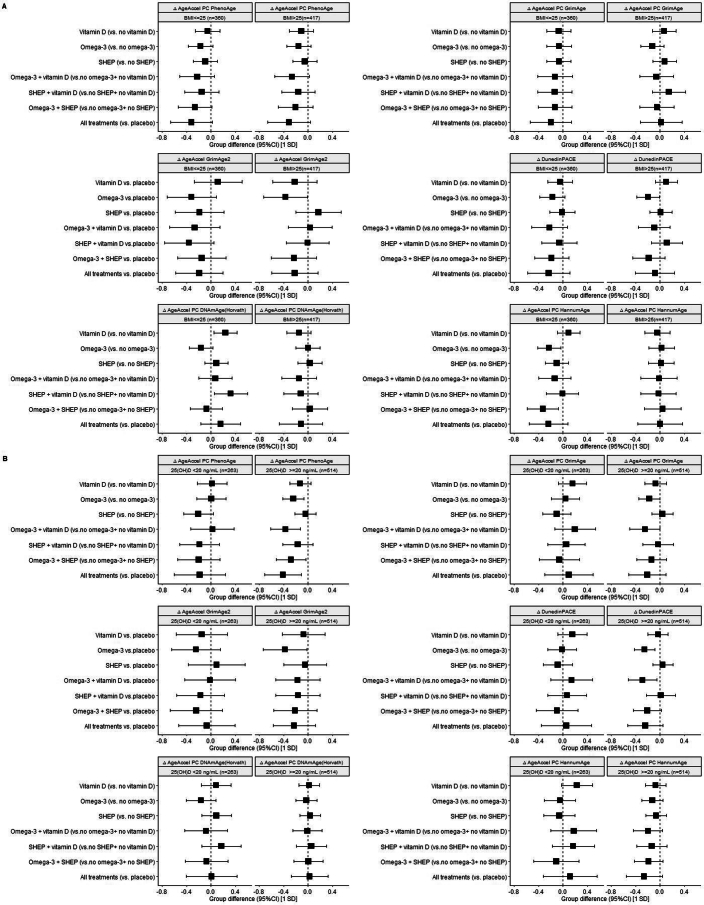
Stratified treatment effects of vitamin D, omega-3 and SHEP (simple home exercise program) individually and in combination on change of DNAm measures from baseline to year 3. Panels **A** and **B** show regression coefficients, and their 95% CI derived from analysis of covariance adjusted for chronological age (continuous + spline at 85 years), sex, falls before study, body mass index, study site, respective baseline biological age measure. All analyses were done in n = 777 participants sampled at baseline and at 3-year follow-up, without technical replicates. The treatment effects in Panel A are stratified by BMI (≤25 kg/m2 vs >25 kg/m2) and in Panel B by baseline serum Vitamin D concentrations (<20 ng/mL vs ≥20 ng/mL).

**Extended Data Fig. 5:**
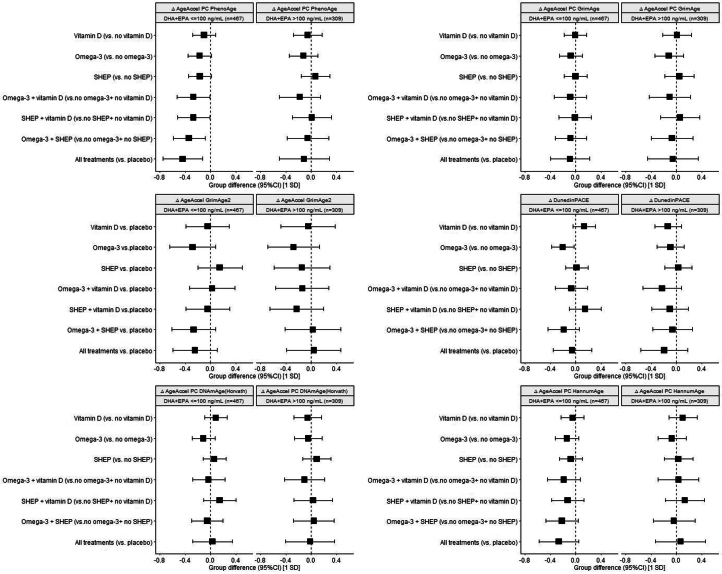
Stratified treatment effects of vitamin D, omega-3 and SHEP (simple home exercise program) individually and in combination on change of DNAm measures from baseline to year 3. The forest plot shows regression coefficients stratified by serum omega-3 concentration (≤100 ng/mL and >100 ng/mL), and their 95% CI derived from analysis of covariance adjusted for chronological age (continuous + spline at 85 years), sex, falls before study, body mass index, study site, respective baseline biological age measure. All analyses were done in n = 777 participants sampled at baseline and at 3-year follow-up, without technical replicates.

As predefined in the study protocol, we investigated whether treatment effects exhibited different patterns based on sex, age (70–74 versus 75+ years), BMI (≤25 versus >25 kg m^−2^ at baseline), baseline 25(OH)D levels (<20 versus ≥20 ng ml^−1^) and baseline polyunsaturated fatty acid levels (≤100 versus >100 ng ml^−1^). The effects of omega-3 supplementation on PhenoAge were somewhat larger for individuals with a baseline 25(OH)D level of ≥20 ng ml^−1^. In addition, the additive effects of combinations of treatments on PhenoAge were somewhat larger for women and individuals with lower baseline blood levels of the omega-3s docosahexaenoic acid (DHA) and eicosapentaenoic acid (EPA). Stratified treatment effects are reported in Extended Data Figs. 3–5.

## Discussion

Previously in DO-HEALTH (including all 2,157 participants), we reported that omega-3 alone reduced the rate of infections by 13% (ref. ^22^) and the rate of falls by 10% (ref. ^23^), and all three interventions combined showed a significant additive benefit on reducing prefrailty by 39% (ref. ^24^) and incident invasive cancer by 61% (ref. ^25^) over a 3-year follow-up. The aim of the DNAm analysis in DO-HEALTH Bio-Age was to assess the effects of the interventions at the molecular level. Three of the four DNAm measures showed the clearest signal for omega-3, highlighting a specific and notable epigenetic response. This specificity is encouraging and supports the idea that targeted nutritional strategies can have distinct epigenetic aging effects. Moreover, the observation that individuals with lower starting levels of omega-3 exhibited larger epigenetic shifts further strengthens the case for personalized approaches. This suggests that baseline nutritional status may modulate the extent of epigenetic responsiveness, emphasizing the potential of omega-3 as a targeted intervention to influence DNAm age and, by extension, biological aging. Furthermore, the PhenoAge findings indicate additive benefits of omega-3 with vitamin D and exercise, extending the evidence from incident invasive cancer and prefrailty in DO-HEALTH to the molecular level. Additional support for an additive benefit of the three interventions comes from four of the seven GrimAge-based epigenetic biomarkers examined in our study (DNAm PAI-1, B2M, TIMP-1 and GDF-15).

Similar to recent data from the CALERIE trial^9^, the treatment effects in DO-HEALTH Bio-Age varied across the DNAm measures of aging we analyzed. Across the epigenetic clocks included in our analyses, the effect was the strongest in the second-generation clocks and DunedinPACE, consistent with evidence of lifestyle effects on these clocks from prior observational studies^5,9,30,31^. In the CALERIE trial, caloric restriction slowed the pace of aging as measured by DunedinPACE but did not affect PhenoAge, GrimAge or the first-generation clocks^9^. In DO-HEALTH, we documented consistent effects of omega-3 treatment across PC-PhenoAge, GrimAge2 and DunedinPACE, as well as in three of the seven GrimAge plasma proteins (PAI-1, leptin and TIMP-1). We also documented an additive effect of the three treatments (omega-3, vitamin D and exercise) on PC-PhenoAge and four of the seven GrimAge plasma proteins (PAI-1, B2M, TIMP-1 and GDF-15).

The CALERIE intervention induced a 2–3% reduction in the pace of aging as measured by DunedinPACE. The effect of the DO-HEALTH interventions on DunedinPACE was somewhat more modest (about a 1% reduction in the pace of aging). However, the reductions in PhenoAge and GrimAge2 by 2.9–3.8 months over 3 years were larger. Further, even small changes in biological aging, if sustained, may have relevant effects on population health^32–34^.

We acknowledge the limitations of our study. There is no gold standard measure of biological aging^35^. We analyzed the best-validated epigenetic clocks, which represent the current state of the art in the field. Rather than specifying a single primary outcome variable, we focused on the consistency of findings across multiple well-validated measures of biological aging, concentrating on second-generation clocks and DunedinPACE^36^. We recognize that DNAm measurements provide only a partial view of the biological changes associated with aging and come with certain technical limitations^37,38^. Moreover, our analysis could make use of only two time points of data: at baseline and the 3-year follow-up. Relying on the change measured across two time points will increase the measurement error relative to three or more time points^39^. However, these limitations should bias effect estimates toward the null, making our estimates of treatment effects conservative. Further, DO-HEALTH ran for only 3 years. Therefore, the significance of the intervention effects on the clocks for long-term survival is unknown^8^. Whether the DO-HEALTH treatments resulted in a persistent slowing of biological aging, leading to the prevention or delay of frailty and chronic disease beyond the 3-year follow-up, is currently unknown. For comparative context, recent meta-analyses report a 3–6% reduction in all-cause mortality risk with vitamin D supplementation^40–43^. Estimates from meta-analyses on the effect of omega-3 fatty acid supplementation on mortality are less comparable to data from DO-HEALTH, as those studies typically involved individuals at high cardiovascular risk with a reduction in mortality risk of about 3–4% (refs. ^44–49^).

The epigenetic clocks we analyzed in DO-HEALTH were developed to measure aging at the organism level and do not specify which organ systems may have been affected by the intervention^50^. However, all three DO-HEALTH interventions were chosen to have diverse protective effects across multiple organ functions. Therefore, the outcome measures we analyzed are well aligned with the intervention.

The DO-HEALTH trial sample does not reflect the general population of adults aged 70 years and older. DO-HEALTH preselected generally healthy and active adults aged 70 years and older; within the Swiss subgroup, more than 50% met the Nurses’ Health Study criteria for healthy agers at baseline. Our subgroup analyses suggested a stronger benefit of omega-3 in participants who started with lower baseline omega-3 (DHA plus EPA) blood levels. Notably, the Swiss subgroup had lower baseline blood omega-3 levels than the total DO-HEALTH population, possibly reflecting the landlocked geography of Switzerland (Extended Data Table 1), which may have supported the benefit of omega-3 supplementation on biological aging in this subsample of DO-HEALTH.

Our findings advance geroscience in two ways. First, similar to the analysis of the pioneering CALERIE trial^9^, we used epigenetic clocks to analyze the effects of interventions implemented in a randomized controlled trial designed to modify biological aging. Prior analyses of DO-HEALTH including all 2,157 participants established that the interventions had additive protective effects on incident invasive cancer^25^ and incident prefrailty^24^. Therefore, the positive findings for the three best-validated epigenetic clocks analyzed in our study support the geroscience hypothesis that slowing biological aging can contribute to preventing chronic diseases^51^. Second, our study bolsters the evidence from a randomized controlled trial for omega-3 as a single intervention and the additive benefit of a combined treatment of omega-3 and vitamin D and exercise on epigenetic clocks, especially PhenoAge. Several articles detail the processes for qualifying and validating biomarkers of biological age^52,53^. Ideally, these measures of biological age can serve as surrogates for primary clinical outcomes. This requires demonstrating that a biomarker responds to rejuvenation therapies and mediates the link between such therapies and clinical outcomes. Consequently, data from human clinical trials, including those from the current study, are especially valuable for validating biological age indicators. However, for many epigenetic biomarkers, the underlying biological mechanisms remain incompletely understood. Finally, based on a recent publication suggesting that epigenetic age is higher at noon than at midnight^54^, we note that blood sampling in DO-HEALTH was standardized to take place in the morning and in a fasting state.

In sum, our analysis provides evidence supporting the geroprotective benefits of omega-3 supplementation and also suggests the benefits of additive combinations of omega-3 supplementation with vitamin D supplementation and exercise. It also motivates further analysis of the DO-HEALTH trial. DO-HEALTH collected biospecimens at additional time points between baseline and the 3-year follow-up and included participants from four other countries in Europe. Results from our study motivate further investigation of these additional biospecimens and participants. More broadly, our findings support the application of measurements of biological aging in general and later-generation epigenetic clocks in particular in the evaluation of interventions designed to slow aging and increase health span.

## Methods

We conducted new DNAm assays of stored blood biospecimens collected from the DO-HEALTH randomized controlled trial (ClinicalTrials.gov identifier: NCT01745263) and merged these data with existing clinical data from the trial. Biospecimen assays were conducted blinded to the trial interventions and outcomes in the subgroup of 777 Swiss participants with samples available at baseline and year 3. The Cantonal Ethical Committee of the Canton of Zurich approved this study (BASEC-Nr 2021-02510). Written informed consent was obtained from all participants included in the study.

### Study design and participants

This randomized, double-blind, placebo-controlled trial with a 2 × 2 × 2 factorial design had three primary treatment comparisons: (1) 2,000 IU per day of vitamin D compared to placebo; (2) 1 g per day of omega-3s (330 mg EPA plus 660 mg DHA from marine algae) compared to placebo; and (3) a strength-training exercise program performed for 30 min three times per week compared to an attention control exercise program focused on joint flexibility performed for 30 min three times a week. The factorial design was chosen to evaluate both the main and combined effects of the interventions. The inclusion criteria were age 70 years and older, living at home, having no major health events (no cancer or myocardial infarction) in the 5 years before enrollment, having sufficient mobility to visit the study centers without help and having good cognitive function with a Mini-Mental State Examination score of at least 24 (refs. ^22,26^). Participants did not receive any compensation for their involvement in the trial. The study and biobank are described at https://do-health.eu.

### Randomization and masking

After enrollment and baseline testing, participants were randomized to one of eight treatment groups (Fig. 1) using block randomization (block sizes of 16 individuals) stratified by recruitment center, prior falls, sex and age (70–84 years or ≥85 years). A central randomization center in Switzerland, supported by trial software, was responsible for the blinding, treatment allocation and study intervention labeling. Participants received two gel capsules per day (vitamin D or placebo and omega-3s or placebo), identical in size, appearance, taste and weight. All capsules had coatings to prevent unblinding by aftertaste^22,26^.

### Procedures

Participants were followed up for 3 years, both with yearly clinical visits (baseline and 1, 2 and 3 years) and telephone calls every 3 months. For DO-HEALTH Bio-Age, the subgroup of 777 Swiss DO-HEALTH participants was assessed at baseline and 3 years. The trial was performed at seven centers in five countries (Switzerland, France, Germany, Portugal and Austria). The study protocol and statistical analysis plan were approved by ethics and regulatory agencies in all five countries and have been previously published^22,26^. A data and safety monitoring board oversaw the study. Adherence to study medication was high, with 86% of the participants taking at least 80% of their total study pills; 70% of the participants performed the exercise programs at least twice per week, and 62% performed the exercise programs at least three times per week. High adherence to study medications (omega-3 and vitamin D supplements) was confirmed by changes in serum polyunsaturated fatty acid and 25(OH)D levels^22,26^.

### DNAm data

Whole blood samples from study participants were collected in PAXgene DNA tubes and registered at the DO-HEALTH Biobank of the University of Zurich. Blood aliquots were sent to the Life&Brain Center, Department of Genomics, University of Bonn, on dry ice for DNA extraction with a chemagic magnetic beads-based method. DNA aliquots were processed on the Infinium Methylation EPIC version 1.0 array (Illumina). The EPIC array quantifies 5-methylcytosine levels at >850,000 CpG sites across all known genes, regions and key regulatory regions. Briefly, a 500 ng amount of extracted DNA samples was bisulfite converted using the EZ-96 DNAm lightning kit (Zymo Research), and 200 ng of the converted DNA was used as input for the EPIC arrays (Illumina). EPIC arrays were processed according to the manufacturer’s instructions and scanned using the Illumina iScan platform. The baseline and 36-month samples from the same individual were processed in the same array batch and on the same BeadChip to minimize batch effects. Quality control and normalization analyses were performed using the minfi (version 1.42.0) Bioconductor (version 2.46.0)^55^ package for the R statistical programming environment (version 3.6.3). Probes were considered missing in a sample if they had detection *P* values of >0.01 and were excluded from the analysis if they were missing in >50% of the samples. Normalization to eliminate systematic dye bias in two-channel probes was carried out using the minfi default method. Following quality control and normalization, DNAm data for 866,238 CpGs were available for 777 participants of the Swiss subgroup in DO-HEALTH, both at baseline and 3 years of follow-up (Fig. 1). Beta values were extracted and used for the analysis.

### Epigenetic clocks and DNAm-based protein measures

Epigenetic clocks are DNAm algorithms that combine information from measurements across the genome to quantify variation in biological aging^1^. The first-generation epigenetic clocks were developed by comparing DNAm between individuals of different chronological ages to generate age prediction algorithms. We report analyses of two well-known first-generation clocks, Horvath’s multitissue clock^2^ and the blood-based clock developed by Hannum et al.^3^. These clocks are highly accurate in predicting chronological age but show only weak and inconsistent associations with morbidity and mortality^2,3,7^. The second-generation epigenetic clocks were developed by modeling differences in mortality risk and normalizing predicted risks to age values. We analyzed two second-generation clocks: PhenoAge^4^ and GrimAge2 (ref. ^8^). These second-generation clocks have much greater predictive capacity for morbidity and mortality than the first-generation clocks^56^.

The DunedinPACE pace-of-aging clock^6^ models differences in the rate of deterioration in organ system integrity, termed ‘pace of aging’ (ref. ^57^). In contrast to first- and second-generation DNAm clocks, which aim to quantify the amount of biological aging at the time of measurement, pace-of-aging clocks quantify the pace of age-related deterioration of system integrity. The DunedinPACE DNAm algorithm was derived from elastic net regression of the physiological pace-of-aging composite on Illumina EPIC array DNAm data derived from blood samples collected at the follow-up assessment at age 45 years. The CpG sites included in the DNAm dataset used to develop the DunedinPACE algorithm were restricted to those showing acceptable test–retest reliability as determined in the analysis by Sugden et al.^58^. The DunedinPACE DNAm algorithm is described in detail in the paper by Belsky et al.^6^.

For the Horvath, Hannum, PhenoAge and GrimAge clocks and the DNAm-based proteins underlying the GrimAge clock, we analyzed versions constructed from DNAm PCs, which have superior technical reliability compared to the original versions of these measures. This was achieved using the computational method by Higgins-Chen et al.^29^. The original versions of GrimAge2 and DunedinPACE demonstrate strong technical reliability^6^. Optimal test–retest reliability is a critical feature of measurements used to evaluate the impact of an intervention.

To compute GrimAge2, we submitted selected CpGs to the DNAm clock calculator hosted by the Horvath laboratory (https://dnamage.genetics.ucla.edu/home; last accessed March 25, 2024) to derive the five DNAm clock estimates and DNAm protein estimates. After processing by the clock calculator (*n* = 790; Fig. 1), a complete sample of *n* = 777 with baseline and follow-up measurements was available for analysis.

The PC versions of the DNAm clocks (except GrimAge2) were calculated using the R code hosted on GitHub (https://github.com/MorganLevineLab/PC-Clocks)^29^ with R (version 4.2.1).

DunedinPACE was calculated for the same samples as the other DNAm variables according to the method described by Belsky et al.^6^, using the R code hosted on GitHub (https://github.com/danbelsky/DunedinPACE/) with R (version 4.2.1).

We first calculated the residuals of the regression of chronological age and biological age and then computed the difference between baseline and year 3. This difference was standardized (mean = 0, s.d. = 1) and was the outcome of our trial (Δ_year 3−baseline_).

### Analysis

The analysis included all Swiss DO-HEALTH participants with available DNAm data at the trial baseline and the 3-year follow-up. We computed standardized change scores for all DNAm measures by comparing standardized residuals at the 3-year follow-up to those at baseline. We analyzed these change scores to test the hypothesis that omega-3 and/or vitamin D and/or exercise slow biological aging by conducting an intention-to-treat analysis comparing the change scores between participants randomized to the interventions and the control groups. We used analysis of covariance models for each of the DNAm measures. If there was no interaction between treatments, we quantified treatment effects as ‘main effects’ (for example, receiving vitamin D versus not receiving vitamin D while adjusting for other treatments). Alternatively, in the case of interactions between treatments, each treatment arm was compared to the placebo arm. The models were adjusted for chronological age (continuous and spline at 85 years), sex, history of falls before study enrollment (a stratifying variable of the trial), BMI and study site. The primary predictors were the three treatments and their interaction with time. Given multiple testing across four second-generation clocks, we focused on identifying consistent patterns in which changes from baseline to follow-up at year 3 had 95% CIs that did not include zero.

### Standardization of effect sizes

Standardization (or normalization) is useful because it allows the comparison of epigenetic biomarkers measured on different scales by placing them on a common scale.

The SAS code below standardizes the change scores (differences) for the specified variables from baseline to the 3-year follow-up. By standardizing, each measure of epigenetic age acceleration (EAA) will have a mean of 0 and an s.d. of 1. We used the PROC STANDARD procedure in SAS to standardize several measures of difference in EAA (residuals of chronological versus biological age). The following SAS code was used for this standardization:

PROC STANDARD DATA = eaa_data MEAN = 0 STD = 1 OUT = df2;

VAR d_EAA d_EAApheno d_EAAhannum d_EAAgrim d_eaagrim2 d_PoA;

RUN;

In this code, the d_ prefix indicates the difference between the follow-up and baseline measures. This standardization process ensures that each difference measure has a mean of 0 and an s.d. of 1, facilitating easier comparison and analysis.

Explanation:

1. DATA = eaa_data. This specifies the input dataset named eaa_data. This dataset contains the variables that will be standardized.
2. MEAN = 0 STD = 1. These options indicate that the standardized variables should have a mean of 0 and an s.d. of 1.
3. OUT = df2. This specifies the name of the output dataset, which, in this case, is df2. The standardized variables will be stored in this new dataset.
4. VAR. This statement lists the variables to be standardized. The variables in this code are listed below.
  d_EAA
  d_EAApheno
  d_EAAhannum
  d_EAAgrim
  d_eaagrim2
  d_PoA

The d_ prefix stands for ‘difference’, indicating that these variables represent the difference (or change) from baseline to the 3-year follow-up. For example, d_EAA = EAA.followUp − EAA.baseline.

### Baseline descriptives

We analyzed data from all Swiss DO-HEALTH participants for whom blood DNAm data were available at baseline and the 3-year follow-up (*n* = 777). The participants had a mean age of 75.5 years (s.d. = 4.5 years), and 60% were women (Table 1). Overall, 52% met the Nurses’ Health Study definition of healthy agers; the baseline average 25(OH)D level was 23.6 ng ml^−1^ (s.d. = 8.4 ng ml^−1^); and the baseline blood omega-3 (DHA and EPA) levels were, on average, 94.3 ng ml^−1^ (s.d. = 40.1 ng ml^−1^). The average baseline BMI was 25.7 kg m^−2^ (s.d. = 4.0 kg m^−2^), and 88% were physically active (29% were active one to three times per week, and 59% were active more than three times per week) based on the well-validated Nurses’ Health Study physical activity questionnaire^59^. Extended Data Table 1 shows the comparison of the Swiss subset to the total DO-HEALTH sample and the Swiss participants who had incomplete DNAm data and thereby were dropped from this analysis.

### DNAm clocks

For the Horvath, Hannum and PhenoAge clocks, we analyzed versions constructed from DNAm PCs, which have superior technical reliability compared to the original versions of these measures^29^. The original versions of the GrimAge2 and DunedinPACE clocks already demonstrate high technical reliability^5,6,8^. All clocks except for DunedinPACE were regressed on chronological age, and residual values were calculated for analysis. DNAm clocks estimate biological age with regard to an organism’s biological state in comparison to a reference population age in which this state would be typical (Extended Data Table 5).

#### PhenoAge clock

The PhenoAge clock is another DNAm-based biomarker developed based on the analysis of nine blood chemistry markers, age and mortality data from the US National Health and Nutrition Examination Surveys (*n* = 9,926 participants aged 18 years and older; 23 years of mortality follow-up); DNAm and blood chemistry data from the InCHIANTI (Invecchiare in Chianti) study (*n* = 912 participants aged 21–100 years); and data from the US Health and Retirement Study (*n* = 3,593 participants aged 51–100 years)^4^.

#### GrimAge version 1 clock

The GrimAge clock was developed as a composite biomarker of seven DNAm surrogates of seven plasma proteins^5^, a DNAm-based estimator of smoking pack-years, age, and sex in the Framingham Heart Study Offspring and Gen3 cohorts (*n* = 2,751 participants aged 24–92 years)^5^. GrimAge relies on the fact that some (but not all) plasma protein levels can be estimated based on cytosine methylation levels. We included in our analyses the seven DNAm-based GrimAge proteins that relate to kidney function, mitochondrial function, blood clotting and inflammation (Extended Data Fig. 1).

#### GrimAge version 2 clock

The same unique 1,030 CpGs were used to construct version 2 of GrimAge based on individuals aged between 40 and 92 years who contributed 13,399 blood samples across nine study cohorts^8^. GrimAge2 outperforms GrimAge version 1 in predicting mortality across multiple racial/ethnic groups and in terms of associations with age-related conditions such as coronary heart disease^8^.

#### DunedinPACE

Pace-of-aging measures estimate the rate of biological aging, defined as the rate of decline in overall system integrity. Pace-of-aging values correspond to the years of biological aging experienced during a single calendar year. A value of 1 represents the typical pace of aging in a reference population; values greater than 1 indicate a faster pace of aging; values lower than 1 indicate a slower pace of aging. Based on the analysis of the pace of aging in the Dunedin Study (*n* = 817 participants examined at ages 26, 32, 38 and 45 years), the pace of aging was measured from within-person change over time in 19 blood chemistry and organ function test metrics of system integrity^6^. DNAm was measured at age 45 years.

### Statistics and reproducibility

No formal sample size calculation was performed specifically for this study. The Swiss National Science Foundation funded DNAm assays of samples collected at baseline and 36 months from the Swiss subset of DO-HEALTH participants. The study flowchart is presented in Fig. 1. Of the 1,006 Swiss participants, 790 provided consent for these analyses. Samples with insufficient DNA extraction for accurate DNAm measures and samples that showed a mismatch of predicted sex based on DNAm measures and reported sex were excluded. This resulted in a sample size of *n* = 777 participants. A preliminary power calculation, based on the number of participants with both baseline and year 3 blood samples and consent, indicated that this sample size would provide 90% power for detecting the anticipated effects. No technical replicates were run for the DNAm analysis of the EPIC array.

### Reporting summary

Further information on research design is available in the Nature Portfolio Reporting Summary linked to this article.

## Supplementary information

Reporting Summary

## Figures and Tables

**Extended Data Table 1:**
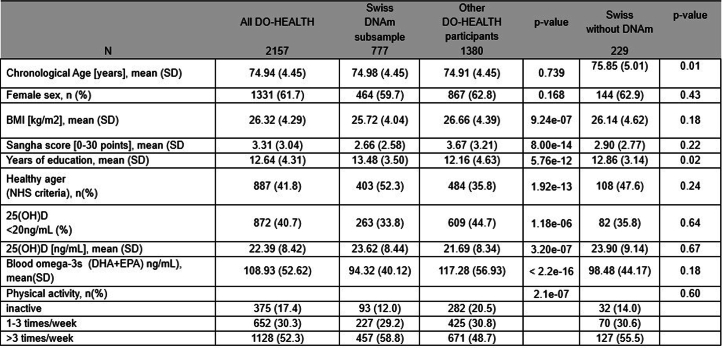
Comparison of baseline characteristics of the Swiss subgroup with and without DNAm measures and other participants of DO-HEALTH

**Extended Data Table 2:**
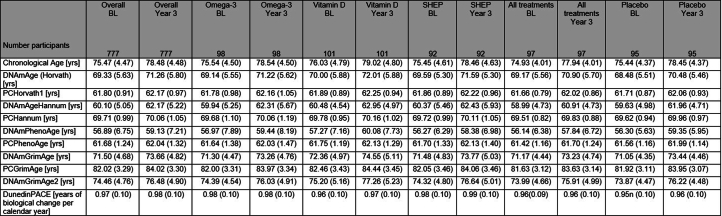
Mean values of the DNAm measures of aging for omega-3 alone, vitamin D alone, SHEP (simple home exercise program) alone, the combination of all three treatments and placebo at baseline and 36-month follow-up

**Extended Data Table 3:**
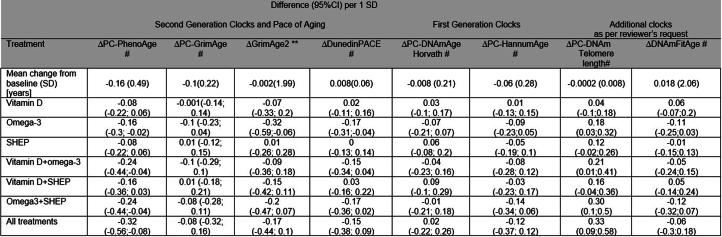
Treatment effects of vitamin D, omega-3 and SHEP (simple home exercise program) individually and in combination on the change of DNAm measures from baseline to year 3

Treatment effects are expressed as standardized estimates of the change in DNAm measure from baseline to year three at the respective 95%CI. All analyses show main effects under the assumption of additive effects between treatments in the 2x2x2 factorial trial design, that is comparing all individuals treated with vitamin D across the 8 treatment arms of the trial compared with those who did not receive vitamin D (same for omega-3 and SHEP compared to control SHEP). Only for GrimAge2 each treatment arm is compared to placebo, because of treatment interactions. There is consistency for omega-3 slowing biological aging as measured by PhenoAge, GrimAge2 and Pace of Aging. PhenoAge additionally shows the additive benefit between treatments. There was no significant treatment effect on the change in DNAmFitAge^61^ over three years. Additionally, the combination of Vitamin D and omega-3 (0.21(0.01;0.41)), the combination of SHEP and omega-3 (0.30 (0.1;0.5)), as well as the combination of all three treatments (0.33 (0.09;0.58)) significantly increases PC-DNAmTL over three years. DNAmTL it is not a good proxy for telomere length (weak correlation of around 0.35). However, DNAmTL has a stronger relationship to age, smoking, obesity, mortality risk) than actual leukocyte telomere length based on Southern blotting or PRC measures^62^. The table shows regression coefficients, and their 95% CI derived from analysis of covariance adjusted for chronological age (continuous + spline at 85 years), sex, falls before study, body mass index, study site, respective baseline biological age measure. All analyses were done in n = 777 participants sampled at baseline and at 3-year follow-up, without technical replicates

To derive the effect of each treatment in month the respective estimate needs to be multiplied by the standard deviation and by 12. To translate effect units to months: (Standardized estimate* SD + mean)*12; that is (−0.16*0.49-0.16)*12* = 2.9 months; (−0.32*0.49-0.16)*12 = −3.8 months. See Fig. 2 – visual display of the same findings.

**Extended Data Table 4:**
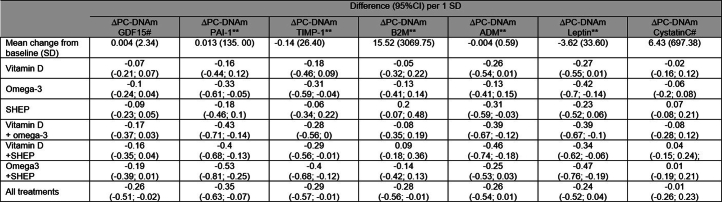
Treatment effects of vitamin D, omega-3 and SHEP (simple home exercise program) individually and in combination on change of DNAm-based surrogate biomarkers of plasma proteins based on GrimAge

The seven DNA methylation based estimators of plasma proteins estimate plasminogen activation inhibitor 1 (DNAm PAI-1), beta‐2 microglobulin (DNAm B2M), adrenomedullin (DNAm ADM), leptin (DNAm Leptin), tissue inhibitor metalloproteinase 1 (DNAm TIMP‐1), growth differentiation factor 15 (DNAm GDF‐15), cystatin C (DNAm Cystatin C). For the seven DNAm-based proteins, omega-3 stands out as an individual treatment with a decline in three of seven DNAm-based surrogate biomarkers of plasma proteins (PAI-1, Leptin, TIMP-1). However, the data also shows consistency of an additive benefit combining two or all three treatments for several plasma proteins (PAI-1, B2M, TIMP-1, GDF-15) See Fig. 3 – visual display of the same findings. The table shows regression coefficients, and their 95% CI derived from analysis of covariance adjusted for chronological age (continuous + spline at 85 years), sex, falls before study, body mass index, study site, respective baseline DNAm surrogate marker. All analyses were done in n = 777 participants sampled at baseline and at 3-year follow-up, without technical replicates.

**Extended Data Table 5:**
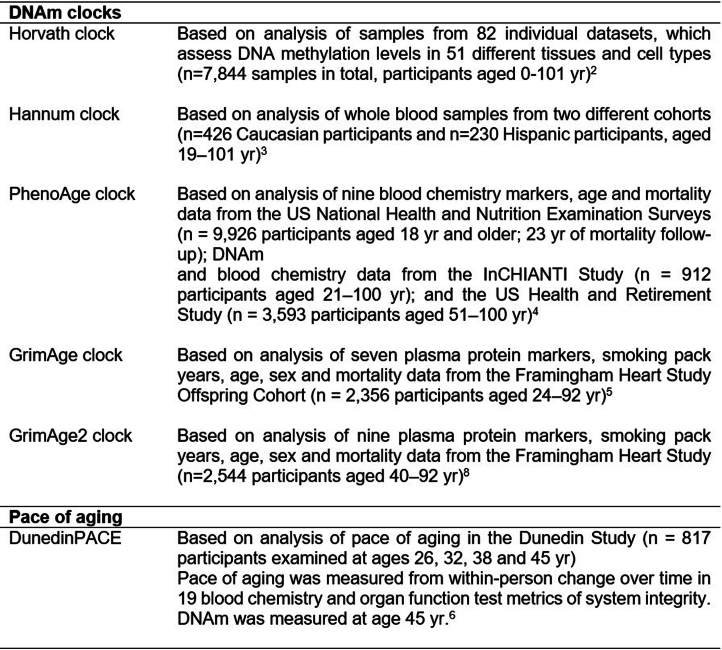
DNAm clocks and pace-of-aging measures included in the DO-HEALTH analysis

## Data Availability

Data from DO-HEALTH, used in the context of this project, as well as codebooks and analytic codes, will initially be reserved for the primary researchers of the Center of Aging and Mobility Research Group to fully exploit the datasets. Subsequently, the data will be made available to external researchers according to a controlled access system. However, all data supporting the findings of this study are available from the corresponding author upon request.

## Code availability

Previously published code was used in this study^6,29^, and no new code was generated.
