# Supplementary material for: Individual and additive effects of vitamin D, omega-3 and exercise on DNA methylation clocks of biological aging in older adults from the DO-HEALTH trial
Source: Nat Aging. 2025 Feb 3;5(3):376–85. doi: 10.1038/s43587-024-00793-y (PMC11922767; doi:10.1038/s43587-024-00793-y)
Supplement: Supplementary file 1 — Reporting Summary [file 43587_2024_793_MOESM1_ESM.pdf]

nature portfolio

Corresponding author(s): Heike A. Bischoff-Ferrari

Last updated by author(s): Nov 5, 2024

Reporting Summary

Nature Portfolio wishes to improve the reproducibility of the work that we publish. This form provides structure for consistency and transparency in reporting. For further information on Nature Portfolio policies, see our Editorial Policies and the Editorial Policy Checklist.

Statistics

For all statistical analyses, confirm that the following items are present in the figure legend, table legend, main text, or Methods section.

n/a	Confirmed	
☐	☒	The exact sample size (*n*) for each experimental group/condition, given as a discrete number and unit of measurement
☐	☒	A statement on whether measurements were taken from distinct samples or whether the same sample was measured repeatedly
☐	☒	The statistical test(s) used AND whether they are one- or two-sided<br>*Only common tests should be described solely by name; describe more complex techniques in the Methods section.*
☐	☒	A description of all covariates tested
☐	☒	A description of any assumptions or corrections, such as tests of normality and adjustment for multiple comparisons
☐	☒	A full description of the statistical parameters including central tendency (e.g. means) or other basic estimates (e.g. regression coefficient) AND variation (e.g. standard deviation) or associated estimates of uncertainty (e.g. confidence intervals)
☐	☒	For null hypothesis testing, the test statistic (e.g. F, t, r) with confidence intervals, effect sizes, degrees of freedom and P value noted<br>*Give P values as exact values whenever suitable.*
☒	☐	For Bayesian analysis, information on the choice of priors and Markov chain Monte Carlo settings
☒	☐	For hierarchical and complex designs, identification of the appropriate level for tests and full reporting of outcomes
☐	☒	Estimates of effect sizes (e.g. Cohen's *d*, Pearson's *r*), indicating how they were calculated

Our web collection on statistics for biologists contains articles on many of the points above.

Software and code

Policy information about availability of computer code

Data collection	Software for data collection was custom made and provided by Ferrari Data Solutions.
Data analysis	Data analysis was performed with R (version 4.2.1) and SAS (version 9.4).<br>Quality control and normalization analyses were performed using the minfi (v.1.42.0) Bioconductor (v.2.46.0)55 package for the R statistical programming environment (v.3.6.3).<br>To compute GrimAge2 and the DNAm-based protein estimates included in the GrimAge clock, selected CpGs were submitted to the DNA methylation clock calculator hosted by the Horvath Lab (https://dnamage.genetics.ucla.edu/home; 19.03.2024) to derive the five DNAm clock estimates and DNAm protein estimates. PC versions of the PhenoAge, Horvath, and Hannum epigenetic clocks were computed for the same samples as the GrimAge DNAm proteins according to the method described by Higgins-Chen et al.29 using the R code hosted on GitHub (https://github.com/MorganLevineLab/PC-Clocks) using R (version 4.2.1).<br>DunedinPACE was calculated for the same samples as the other DNAm variables according to the method described by Belsky et al.6 using the R code hosted on GitHub (https://github.com/danbelsky/DunedinPACE/) using R (version 4.2.1). Additional batch correction was performed by residualizing DNAm measurements for PCs estimated from array control-probe beta values for the four clocks DNAmAge, DNAmHannumAge, DNAmPhenoAge and DNAmGrimAge and its single elements using the code provided by Higgins-Chen et al. on GitHub (https://github.com/MorganLevineLab/PC-Clocks) using R (version 4.2.0).

For manuscripts utilizing custom algorithms or software that are central to the research but not yet described in published literature, software must be made available to editors and reviewers. We strongly encourage code deposition in a community repository (e.g. GitHub). See the Nature Portfolio guidelines for submitting code & software for further information.

Data

Policy information about availability of data

All manuscripts must include a data availability statement. This statement should provide the following information, where applicable:

- Accession codes, unique identifiers, or web links for publicly available datasets
- A description of any restrictions on data availability
- For clinical datasets or third party data, please ensure that the statement adheres to our policy

Data used in the context of this study will initially be reserved for the primary researchers of the Center of Aging and Mobility Research Group to fully exploit the datasets. Subsequently, the data will be made available to external researchers according to a controlled access system. However, all data supporting the findings of this study are available from the corresponding author upon request.
Code used to generate the variables in this study has been published previously as referenced in the text, thus code sharing is not applicable to this study.

Research involving human participants, their data, or biological material

Policy information about studies with human participants or human data. See also policy information about sex, gender (identity/presentation), and sexual orientation and race, ethnicity and racism.

Reporting on sex and gender	We collected the biological sex of participants, which was assigned. All study participants provided written, informed consent. However, as predefined in the study protocol, we tested whether the treatment effects varied by sex. Since the interaction for the main findings was not statistically significant, the findings of the present study apply to all sexes.
Reporting on race, ethnicity, or other socially relevant groupings	No variable related to race, ethnicity or socially relevant grouping have been used in the study.
Population characteristics	Participants had mean age of 75.5 yr (s.d. = 4.5), 60% were women. Overall, 52% met the Nurses' Health Study definition for healthy agers, baseline average 25(OH)D status was 23.6 ng/ml (s.d. = 8.4) and baseline blood omega-3 levels (DHA and EPA) were on average 94.3 ng/ml (s.d. = 40.1). Average baseline BMI was 25.7 kg/m2 (s.d. = 4.0) and 88% were physically active (29% moderately and 59% vigorously), based on the well-validated Nurses' Health Study physical activity questionnaire. More details can be found in Table 1.
Recruitment	DO-HEALTH participants were recruited through mailing lists of retirement authorities, churches, and other community services, posters, flyers, public events, advertisement in newspapers and other media, public events and educational programs and health care. The advertisements contained a contact phone number for each specific recruitment site that potential participants were asked to call for further information (DO-HEALTH telephone hotline – established at each recruitment site).
Ethics oversight	The Cantonal Ethical Committee of the Canton of Zurich approved this study (BASEC-Nr 2021-02510)

Note that full information on the approval of the study protocol must also be provided in the manuscript.

Field-specific reporting

Please select the one below that is the best fit for your research. If you are not sure, read the appropriate sections before making your selection.

☒ Life sciences ☐ Behavioural & social sciences ☐ Ecological, evolutionary & environmental sciences

For a reference copy of the document with all sections, see nature.com/documents/nr-reporting-summary-flat.pdf

Life sciences study design

All studies must disclose on these points even when the disclosure is negative.

Sample size	No formal sample size calculation was performed specifically for this analysis. The Swiss National Science Foundation funded DNAm assays for samples collected at baseline and at 36 months from the Swiss subset of DO-HEALTH participants. Of the 1,006 Swiss participants, 777 provided consent for these analyses and had samples available after applying exclusion criteria. A preliminary power calculation, based on the number of participants with both baseline and year 3 blood samples and consent, indicated that this sample size would provide 90% power for detecting the anticipated effects.
Data exclusions	Data were excluded from the analysis due to lack of follow-up or approval for genetic analyses, low quality of DNA extraction, gender mismatch, and absence of follow-up measurements. More details can be found in Figure 1.
Replication	Due to funding limitations, resources were allocated exclusively to the main analyses, and replication studies were not conducted at this time.
Randomization	After enrollment and baseline testing, participants were randomized to 1 of 8 treatment groups (Figure 1) using block randomization (block sizes of 16 individuals) stratified by recruitment center, prior falls, sex, and age (70-84 years or ≥85 years). A central randomization center in Switzerland, supported by trial software, was responsible for the blinding, treatment allocation, and study intervention labeling.

Blinding	All examinations and assessments were performed by trained and certified study staff using standardized methods. Participants, staff dispensing study pills and collecting outcomes, and data analysts were masked to group assignment. A physiotherapist not involved in the assessments provided instructions on the exercise programs.

Reporting for specific materials, systems and methods

We require information from authors about some types of materials, experimental systems and methods used in many studies. Here, indicate whether each material, system or method listed is relevant to your study. If you are not sure if a list item applies to your research, read the appropriate section before selecting a response.

Materials & experimental systems

n/a	Involved in the study	
☒	☐	Antibodies
☒	☐	Eukaryotic cell lines
☒	☐	Palaeontology and archaeology
☒	☐	Animals and other organisms
☐	☒	Clinical data
☒	☐	Dual use research of concern
☒	☐	Plants

Methods

n/a	Involved in the study	
☒	☐	ChIP-seq
☒	☐	Flow cytometry
☒	☐	MRI-based neuroimaging

Clinical data

Policy information about clinical studies

All manuscripts should comply with the ICMJE guidelines for publication of clinical research and a completed CONSORT checklist must be included with all submissions.

Clinical trial registration	NCT01745263
Study protocol	The study protocol has been published in Bischoff-Ferrari, H. A. et al. Effect of Vitamin D Supplementation, Omega-3 Fatty Acid Supplementation, or a Strength-Training Exercise Program on Clinical Outcomes in Older Adults: The DO-HEALTH Randomized Clinical Trial. JAMA 324, 1855-1868 (2020)
Data collection	The trial was performed at 7 recruitment centers located in 5 European countries: Switzerland (University of Zurich, Basel University Hospital, Geneva University Hospital), France (University of Toulouse Hospital Center), Germany (Charité Berlin), Portugal (University of Coimbra), and Austria (Innsbruck Medical University) between December 2012 and November 2017.
Outcomes	We focus the primary hypothesis testing on three “second-generation” epigenetic clocks developed from analyses of mortality risk (PhenoAge, GrimAge and GrimAge2), and a later-generation epigenetic clock, also described as a “third-generation” clock, developed from analysis of longitudinal change in organ-system integrity, DunedinPACE. In the interest of enabling comparison across studies, we also report results for “first-generation” epigenetic clocks developed from analyses of age differences in DNAm (Horvath, HannumAge).<br>Whole blood samples of study participants were collected in PAXgene DNA tubes and registered at the DO-HEALTH Biobank at the University of Zurich. Blood aliquots were sent to Life&Brain, Dept. of Genomics, Bonn, Germany on dry ice for DNA extraction by chemagic magnetic beads-based method. DNA aliquots were processed on Illuminas Infinium Methylation EPIC v1.0 array (Illumina Inc.). Following quality control and normalization, DNAm data for 866,238 CpGs were available for 777 participants of the Swiss subgroup in DO-HEALTH, both at baseline and 36 months follow-up. Beta values were extracted and used for the analysis.<br>The outcomes of the present study were the change in DNAm measures of biological aging between baseline and 36 months of follow-up. Model outcomes were standardized change scores of age acceleration between 36-month follow-up values and baseline values. Change scores were scaled to a mean of 0 and a standard deviation of 1 so that effect sizes can be interpreted as standardized differences between means.

Plants

Seed stocks	*Report on the source of all seed stocks or other plant material used. If applicable, state the seed stock centre and catalogue number. If plant specimens were collected from the field, describe the collection location, date and sampling procedures.*
Novel plant genotypes	*Describe the methods by which all novel plant genotypes were produced. This includes those generated by transgenic approaches, gene editing, chemical/radiation-based mutagenesis and hybridization. For transgenic lines, describe the transformation method, the number of independent lines analyzed and the generation upon which experiments were performed. For gene-edited lines, describe the editor used, the endogenous sequence targeted for editing, the targeting guide RNA sequence (if applicable) and how the editor was applied.*
Authentication	*Describe any authentication procedures for each seed stock used or novel genotype generated. Describe any experiments used to assess the effect of a mutation and, where applicable, how potential secondary effects (e.g. second site T-DNA insertions, mosiacism, off-target gene editing) were examined.*
